# FOXC2-AS1/FOXC2 axis mediates matrix stiffness-induced trans-differentiation of hepatic stellate cells into fibrosis-promoting myofibroblasts

**DOI:** 10.7150/ijbs.81581

**Published:** 2023-08-06

**Authors:** Liankang Sun, Yue Li, Hao Wang, Xuelian Xiao, Xuenan Luo, Ruida Yang, Jinyan Li, Yifei Ma, Qingguang Liu, Kangsheng Tu, Yu Shi

**Affiliations:** 1 Department of Hepatobiliary Surgery, the First Affiliated Hospital of Xi'an Jiaotong University, Xi'an 710061, China.; 2 Zonglian College of Xi'an Jiaotong University, Xi'an 710061, China.; 3 Department of Oncology, the First Affiliated Hospital of Xi'an Jiaotong University, Xi'an 710061, China.

**Keywords:** Hepatic stellate cells, liver fibrosis, matrix stiffness, FOXC2-AS1, FOXC2, RNA exosome complex

## Abstract

Matrix stiffness is a central modulator of hepatic stellate cells (HSCs) activation and hepatic fibrogenesis. However, the long non-coding RNAs (lncRNAs)-regulated transcriptional factors linking matrix stiffness to alterations in HSCs phenotype are not completely understood. In this study, we investigated the effects of matrix stiffness on HSCs activation and its potential mechanism. Through analysis the RNA-seq data with human primary HSCs cultured on 0.4 kPa and 25.6 kPa hydrogel, we identified that forkhead box protein C2 (FOXC2) and its antisense lncRNA FXOC2-AS1 as the new mechanosensing transcriptional regulators that coordinate HSCs responses to the matrix stiffness, moreover, FOXC2 and FOXC2-AS1 expression were also elevated in human fibrosis and cirrhosis tissues. The matrix stiffness was sufficient to activate HSCs into myofibroblasts, resulting in nuclear accumulation of FOXC2. Disrupting FOXC2 and FOXC2-AS1 level abrogated stiffness-induced activation of HSCs. Further mechanistic studies displayed that stiffness-upregulated lncRNA FOXC2-AS1 had no influence on transcription of FOXC2. FOXC2-AS1 exerted its biological function through maintaining the RNA stability of FOXC2, and protecting FOXC2 mRNA from degradation by RNA exosome complex. Additionally, rescue assays confirmed that reintroduction of FOXC2 in FOXC2-AS1-depleted HSCs reversed the repression of FOXC2-AS1 knockdown on stiffness-induced HSCs activation. In *AAV6-*treated mice fibrotic models, targeting FOXC2 *in vivo* lead to a reduced degree of liver fibrosis. In sum, our study uncovers a reciprocal crosstalk between matrix stiffness and FOXC2-AS1/FOXC2 axis leading to modulation of HSCs mechanoactivation and liver fibrosis, and present *AAV6* shRNA as an effective strategy that targets FOXC2 leading to the resolution of liver fibrosis.

## Introduction

Liver fibrosis is a wound healing process that forms in response to a variety of nosetiologies, including hepatitis virus infection, cholestatic disorders, alcoholic liver disease (ALD), and non-alcoholic steatohepatitis (NASH)^1^. It is featured by massive deposition of extracellular matrix (ECM) mainly produced and secreted by hepatic stellate cells (HSCs) 2, 3. Despite the large clinical burden caused by liver fibrosis or cirrhosis, it is still lack of good therapeutic options, prompting intensive efforts to develop new strategies for treatment of liver fibrosis^4^. In a fibrotic microenvironment, transforming growth factor beta (TGF-β)-dependent signaling plays an important role on driving HSCs trans-differentiation into fibrosis-promoting myofibroblasts^5^. Activated-HSCs facilitate liver fibrosis by synthesis and secretion of cytokines, chemokines, ECM and matrix metalloproteinases^6^, ^7^. Understanding other pathways controlling HSCs activation or trans-differentiation, in addition to TGF-β signaling, may contribute to elucidate novel strategies for targeting this disease.

Recent evidence indicates that matrix stiffness is central to the persistent HSCs activation that drives progression of fibrosis^8^, ^9^. Elevated stiffness is not only the consequence of hepatic fibrosis, but now also considered as a key driver in the pathological progression of liver fibrosis^9^. External matrix stiffness is relayed into intracellular signaling cascades that eventually converge onto transcriptional factors^10^. Previous studies have already found that YAP/TAZ, ZNF416, MKL1/SRF and MRTF-A are highly identified mechanosensing transcriptional regulators mediated stiffness-induced HSCs or fibroblasts activation and fibrosis^10^-^12^. Moreover, epigenetic regulators such as P300 acetyltransferase, a transcription coactivator, also promotes stiffness-induced activation of HSCs into tumor-promoting myofibroblasts via RHOA-AKT signaling^13^. Thus, identifying new mechanosensing transcriptional factors that coordinate HSCs responses to the matrix stiffness is a high priority and may be a novel strategy to conquer liver fibrosis.

Forkhead box protein C2 (FOXC2), a transcription regulator of the forkhead/winged-helix family, plays critical roles on cell proliferation, apoptosis, lymphangiogenesis and differentiation^14^. Previous studies revealed that FOXC2 markedly promotes differentiation of osteoblast via activation WNT-β/catenin signaling^15^, and modulates survival and proliferation of osteoblast through upregulation of integrin β1 as well^16^. FOXC2 senses the oscillatory shear stress, a type of biomechanical force, and maintains endothelial cytoskeleton organization to ensure the postnatal stability of the lymphatic vasculature^17^. Additionally, FOXC2 exerts multiple functions on malignant progression of cancer^14^, ^18^-^20^, for example, FOXC2 nuclear localization inhibits E-cadherin expression, and enhances the expression level of α-SMA, vimentin to promote epithelial-mesenchymal transition (EMT) and metastasis of breast cancer cells^21^. However, the function of FOXC2 in HSCs is still not clear, and urgently needs to be further elucidated.

In this study, through analysis of RNA-seq data of HSCs cultured on polyacrylamide gels with incremental stiffness, we found that matrix stiffness markedly upregulated the expression of FOXC2, and functional experiments elucidated that stiffness-upregulated FOXC2 lead to transcription of α-SMA, Col1A1, CTGF and FN1. Moreover, mechanistic studies revealed that stiffness-induced long non-coding RNA FOXC2-AS1 binds to FOXC2 mRNA and protects it from degradation by the RNA exosome complex to maintain its stability, which plays a crucial role on HSCs activation and liver fibrosis. AAV6 carrying shRNA targeting FOXC2 *in vivo* suppressed CCl4 or bile duct ligation (BDL)-induced HSCs activation and liver fibrosis. Thus, our studies demonstrated that FOXC2 represents a novel target for mitigating stiffness-induced HSCs activation and liver fibrosis.

## 2. Materials and Methods

### 2.1 Cell culture, antibodies and plasmids

The primary human HSCs were purchased from ScienCell (#5300), and cultured in DMEM (Dulbecco's Modified Eagle's Medium) supplemented with 10% fetal bovine serum, streptomycin and penicillin. The monitoring mycoplasma infection of HSCs were performed by utilizing a MycoAlert detection kit (Lonza Group AG, Basel, Switzerland) and these cells were free of infection during the experiments. Human primary HSCs with passage < 8 were utilized for all experiments. The antibodies (anti-α-SMA, anti-Fibronectin, anti-type 1 collagen, anti-CTGF, anti-EXOSC4 and anti-EXOSC10) used in this study were reported as previous described^5^; the anti-FOXC2 (#sc-515234) and anti-cytokeratin 19 (CK-19) (#sc-376126) were purchased from Santa Cruz Biotechnology, Inc. The non-targeting shRNA, shFOXC2-AS1 and full-length FOXC2 overexpression plasmid (pCMV-HA-FOXC2) were constructed by Tsingke Biotech Co (Beijing, China). The shFOCX2 RNA (#sc-43767-SH) were purchased from Santa Cruz Biotechnology, Inc. The shEXOSC10-1: GCCATCGTTAAGGTCTTTCAT; shEXOSC10-2: CGTGGACTCAAACAAGCAAT; shEXOSC4-1: GCCCTAGTGAACTGTCAATAT; shEXOSC4-2: TCAATATAGTTCAGCGACCTT were purchased from MilliporeSigma. The siRNAs targeting FOXC2 (si-FOXC2): 5'-CTACCTGAGCGAGCAGAAT-3', and non-targeting siRNA (si-Control): 5'-TTCTCCGAACGTGTCACGT-3' were obtained from Genepharm (Shanghai, China)^20^.

### 2.2 Human samples of liver fibrosis or cirrhosis

63 human liver fibrosis/cirrhosis specimens were collected from patients undergoing liver transplantation in the First Affiliated Hospital of Xi'an Jiaotong University. Patients who were diagnosed with Wilson's disease, Alpha-1 antitrypsin (AAT) deficiency, or other inherited liver diseases based on standard clinical, laboratory and histological assessments were excluded in this study. 21 normal liver specimens were obtained from patients with liver trauma or hepatic hemangioma. All procedures performed in studies involving human participants were in accordance with the ethical standards of the Research Ethics Committee of The First Affiliated Hospital of Xi'an Jiaotong University and with the 1964 Helsinki declaration and its later amendments. All written informed consent to participate in the study was obtained from patients with liver fibrosis, liver trauma or hepatic hemangioma for samples to be collected from them.

### 2.3 RNA-seq

The generation of RNA-seq data (GSE101343) with primary human HSCs seeded on 0.4 kilopascal (kPa) or 25.6 kPa hydrogels and further bioinformatics analysis was performed as previously reported^5^, ^13^.

### 2.4 Viral packaging and viral transduction of HSCs

Packaging of lentiviruses was performed as we previously described^5^, ^22^. Briefly, the package plasmids (pMD.2G and pSPAX2) and targeted shRNA or pCMV-HA-FOXC2 (with puro resistance) were co-transfected into HEK-293T cells according to the manufacturer's procedure of Effectene® Transfection Reagent (#301425 Qiagen, Germantown, MD). The cells were then cultured for 48 h to secrete virus. The supernatants containing viruses were collected and infected into HSCs for further experiments.

### 2.5 Polyacrylamide hydrogels with different stiffness

Polyacrylamide hydrogels with incremental stiffness with 0.4 kPa or 25.6 kPa were generated according to the protocols as previously reported^11^, ^13^, ^23^.

### 2.6 Immunofluorescence staining (IF)

HSCs cultured on polyacrylamide hydrogels with different stiffness (0.4 kPa or 25.6 kPa) or human liver cryosections were prepared for IF staining, as previously described^5^, ^13^. Briefly, HSCs or human liver cryosections were fixed with 4% PFA (paraformaldehyde) for 15 minutes followed by permeabilization by 0.3% Triton X-100 for 5 minutes. After washing the cryosections with 1 x PBS three times, 5% BSA was administrated to block non-specific antibody binding sites. The cryosections were then placed in primary antibody diluted in blocking buffer and incubated overnight at 4°C. After rinsing with 1 x PBS three times, the samples were then incubated with fluorescent secondary antibody, and DAPI respectively. The samples on the slides were visualized with a Zeiss Instruments confocal microscope (Cal Zeiss AG, Jena, Germany). ImageJ was utilized to quantify the immunofluorescence density of each image.

### 2.7 qRT-PCR

After finishing the designated intervention, the mRNA was extracted from HSCs, human or mice livers by using the Trizol (Thermo Fisher Scientific, California, USA). Equal amounts of mRNA were converted into cDNA (complementary DNA), and quantitative polymerase chain reaction (qPCR) was conducted as we previously described^5^. The primers used for qRT-PCT were displayed in Supplementary Table 1.

### 2.8 Chromatin immunoprecipitation (ChIP) and ChIP-qPCR

HSCs, cultured on 0.4 or 25.6 kPa hydrogels, were harvested for ChIP assay, ChIP assay was conducted using an EZ-Magna-ChIP HiSens kit (Millipore, #17-10461) as previously reported^13^. Briefly, HSCs fixed with 1% formaldehyde and scraped from the hydrogels were subjected to nuclear lysis to release cross-linked protein/DNAs. The nuclear extract was then subjected to sonication and immunoprecipitation with anti-FOXC2 antibody (Abcam, #ab5060) or control rabbit IgG (Santa Cruz Technology, #sc-2027). Precipitated DNA fragments containing the promotor of ACTA2 (α-SMA), CTGF, FN1 and Col1A1 were quantitated by qPCR with the pair of primers. JASPAR software was used to predict the FOXC2 binding with the promoter of HSCs activation markers^24^. The primers for ChIP-qPCR were displayed in Table S2.

### 2.9 Western blot

RIPA buffer (#9806 Cell Signaling Technology) added with a phosphatase inhibitor cocktail (# 78428, Thermo Fisher Scientific) and protease inhibitors (#88266, Thermo Fisher Scientific) was utilized to lyse the hepatic stellate cells or liver tissues. The protein concentration of the samples was assessed by using a BCA protein assay kit (Pierce, Rockford, USA). Western blot assays and densitometric analysis of the designated protein bands were conducted as we previously described^5^, ^25^, ^26^.

### 2.10 RNA-stability analysis

HSCs were incubated with the transcriptional inhibitor actinomycin D (5 µg/mL, Sigma-Aldrich, USA) for 0 h, 2 h, 4 h, and 8 h, respectively. The HSCs were collected at the indicated time points, and RNA was isolated. Then, qRT-PCR was performed to assess the relative mRNA level of FOXC2.

### 2.11 RNA immunoprecipitation (RIP) assay

RIP was conducted by utilizing the EZ-Magna RIP^TM^ RNA Binding Protein Immunoprecipitation Kit (#17-701 MilliporeSigma, Birlington, MA) according to the manufacturer's protocols. Briefly, RIP lysis buffer added with the RNase inhibitors (#3335399001 MilliporeSigma) and protease inhibitor cocktail (#88266 Thermo Fisher Scientific, Waltham, MA) was applied to lyse the cells. Then the specific antibody (anti-EXOSC10 or anti-EXOSC4) and protein A/G magnetic beads were placed in cell lysates for overnight at 4℃. On the second day, the RNA/protein complexes binding on the beads were precipitated by magnetic force, then the RNA was extracted using Trizol and determined by qRT-PCR analysis.

### 2.12 RNA pull-down assay

The FOXC2-AS1 and FOXC2 were *in vitro* transcribed from the recombinant plasmids pCMV3-FOXC2-AS1 or pCMV3-FOXC2, and the Biotin RNA Labeling Mix (Roche, USA) was utilized to generate biotin-labeled FOXC2-AS1 and biotin-labeled FOXC2 RNA as previously reported^19^. Cell lysates were incubated with biotin RNA followed by streptavidin-mediated RNA pulldown. After finishing RNA isolation, qRT-PCR was applied to detect the binding between FOXC2- AS1 and FOXC2.

### 2.13 Subcellular fractionation

The cytosolic and nuclear fractions of hepatic stellate cells were extracted with PARIS Kit (Thermofisher, USA) according to the manufacturer's instructions.

### 2.14 Nascent RNA capture array

Nascent RNA of HSCs was extracted by using the Click-iT Nascent RNA Capture Kit (Life Technologies, USA) following with the manufacturer's instructions. Then, qRT-PCR was performed to examine the relative expression of FOXC2 nascent RNA.

### 2.15 RNA fluorescent in situ hybridization (FISH)

Subcellular localization of FOXC2-AS1 in HSCs was detected by the FISH Kit (RiboBio, Guangzhou, China) following the product's instructions. In brief, HSCs were cultured on coverslips into 24-well plates. Then, the 4% paraformaldehyde was utilized to fix HSCs for 15 minutes. After that, the fixed HSCs were washed with PBS and subjected to permeabilization (0.5%Triton-X PBS for 10 minutes). Subsequently, HSCs were incubated with prehybridization solution and hybridization solution respectively, then hybridized with the cy3-labeled FOXC2-AS1 oligonucleotide probe (RiboBio, Guangzhou, China) overnight at 4℃. HSCs nuclei was visualized with DAPI for 5 min at room temperature. All pictures were taken and recorded by Zeiss Instruments confocal microscope.

### 2.16 CCl4 administration, FOXC2 targeting by AAV6 shRNA and analysis of liver fibrosis

All animal experiments were conducted following the instructions authorized by the ethical committee of Xi'an Jiaotong University. Six- to eight-week male C57BL/6 mice were obtained from Animal Centre of Xi'an Jiaotong University and they were supplied with free access to water and food. Liver fibrosis was induced by intraperitoneal injection of 20% CCl4 (carbon tetrachloride; #289116 Sigma-Aldrich) in olive oil at 5 μL·g-1 of body weight, twice per week. Two weeks after intraperitoneal injection of CCl4, these mice were administered with 100 μL of AAV6-shControl (non-targeting control) or AAV6-shFOXC2 (1.5 × 10^12^ viral genomes·mL-1; Hanbio Biotechnology, Shanghai, China) through tail vein injection, and the detail protocol of *AAV6*-shFOXC2 injection was reported as previously described^27^, ^28^. After an additional 4 weeks' injection of CCl4, the murine HSCs were isolated, and qRT-PCR and western blot were used to confirm the targeting efficiency of *AAV6*-shFOXC2 in murine activated HSCs. The detail protocol for murine HSCs isolation was displayed as previously described^13^. Moreover, the mice livers were harvested and used for following experiments (hydroxyproline analysis, Sirius Red staining, Masson staining, Immunohistochemistry staining, qRT-PCR, western blotting) to evaluate the liver fibrosis. The detailed protocols of hydroxyproline analysis, Sirius Red staining, Masson staining, and Immunohistochemistry staining present as previously described^2^, ^29^.

### 2.17 Bile Duct Ligation (BDL), another mice model for liver fibrosis

BDL was conducted as previously reported^2^. Briefly, six- to eight-week male C57BL/6 mice were underwent either sham or BDL surgery. Mice were anesthetized and the bile duct was ligated using sterile 3/0 silk ligatures. Sham surgery was conducted by opening up the mouse and passing a silk ligature under the bile duct, one week later, these mice were administered with 100 μL of AAV6-shControl or AAV6-shFOXC2 (1.5 × 10^12^ viral genomes·mL-1; Hanbio Biotechnology, Shanghai, China) *via* tail vein injection. After 3 weeks, mice were sacrificed and the livers obtained for further analysis of fibrogenesis, and IHC staining of CK-19 in BDL model was used to show the proliferation of bile duct epithelium^30^.

### 2.18 Statistical analysis

Data were displayed as Mean ± S.E.M. The difference between groups was assessed by Student's t-test or ANOVA followed by a post hoc test applying GraphPad Prism 6 software (GraphPad Software, Inc., La Jolla, CA). *P*<0.05 was considered as statistically different.

## 3. Results

### 3.1 Matrix stiffness induces HSCs activation into myofibroblasts and increases FOXC2 nuclear accumulation

**Figure 1:**
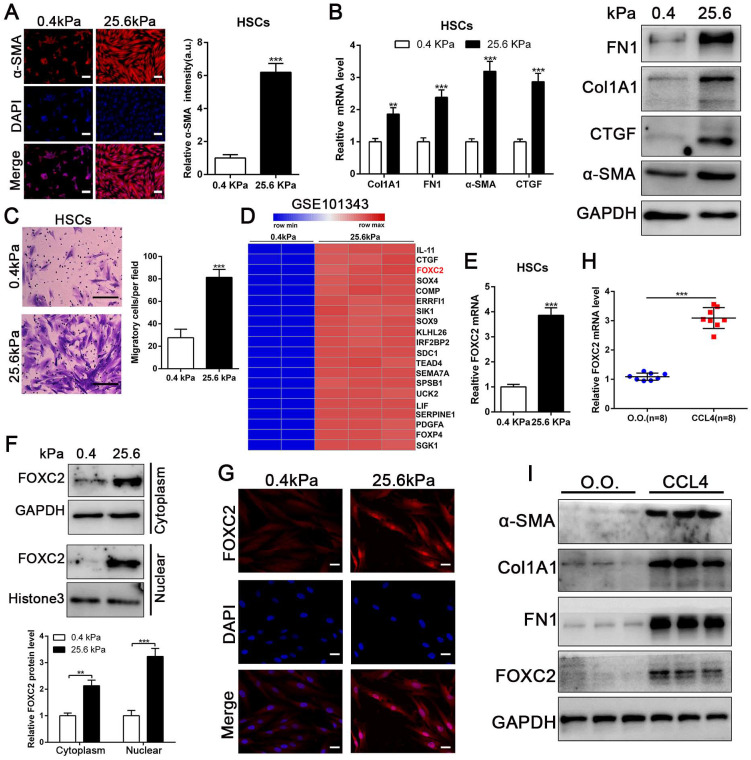
** Matrix stiffness induced HSCs activation, upregulated the expression of FOXC2 and facilitated its nuclear accumulation.** (A-B) Primary human HSCs were plated on polyacrylamide hydrogels and used to perform IF staining, qRT-PCR and WB with quantitative data shown. Stiffness promoted HSCs activation and increased FN1, Col1A1, CTGF and α-SMA expression. Magnification is ×10, and scale bars = 50 μm. n =three independent experiments, **P < 0.01, ***P < 0.001 by Student's t-test versus 0.4kPa. (C) 25.6 kPa stiffness facilitated HSCs migration. The scale bars = 50 μm. n =three independent experiments, ***P < 0.001 by Student's t-test versus 0.4kPa. (D) Analysis of RNA-seq data (GSE101343) present that compared with 0.4kPa, 25.6kPa stiffness greatly induced the expression of a transcription factor, named FOXC2. (E-G) qRT-PCR, subcellular fractionation analysis and IF staining displayed that matrix stiffness effectively induced the significant expression of FOXC2 and promoted the nuclear accumulation of FOXC2. n =three independent experiments, Magnification is ×40, and scale bars = 20 μm.**P < 0.01, ***P < 0.001 by Student's t-test versus 0.4kPa. (H-I) qRT-PCR and WB present that *in vivo* stiffness upregulated the HSCs activation markers and FOXC2 expression as well. n =three independent experiments, ***P < 0.001 by Student's t-test versus O.O. (olive oil).

To evaluate if substrate stiffness in return modulates HSCs activation, we used polyacrylamide hydrogels with precisely defined stiffness as supports for *in vitro* culture of HSCs^13^. The primary human HSCs seeded on a 0.4 kPa or 25.6 kPa hydrogel exhibited obviously different morphology^13^. HSCs cultured on 0.4 kPa hydrogel were unbale to spread and expressed the low level of α-SMA as determined by IF. Conversely, HSCs on 25.6 kPa hydrogel displayed well-spread and increased positive staining for α-SMA, characteristics of activated-HSCs (Fig. 1A). Furthermore, qRT-PCR and western blot also displayed that 25.6 kPa stiffness indeed increased the expression of α-SMA, Col1A1, CTGF and FN1 in comparison to 0.4 kPa (Fig. 1B and Fig.S1A). Accordingly, HSCs cultured on 25.6kPa hydrogel exhibited an increased ability of migration (Fig. 1C). Since stiffness induced fibroblasts activation independent of TGF-β signaling^11^, ^31^, we tried to find the potential mechanism for stiffness-mediated HSC trans-differentiation into myofibroblasts. To this end, we analyzed the RNA-seq data (GSE101343) with HSCs cultured on 0.4 kPa and 25.6 kPa hydrogel, the results displayed that 25.6 kPa stiffness dramatically increased FOXC2 expression level in comparison to 0.4 kPa soft hydrogel (Fig. 1D). FOXC2, a transcription regulator of the forkhead/winged-helix family, plays critical roles on cell proliferation, apoptosis, lymphangiogenesis and differentiation^14^, but its efficacy on matrix stiffness-mediated HSCs activation is not clear. We first performed qRT-PCR and western blot analysis to test if stiffness influenced FOXC2 expression, the data exhibited that 25.6 kPa stiffness notably promoted FOXC2 expression, and concurrently FOXC2 nuclear accumulation in HSCs as compared to 0.4 kPa (Fig. 1E-1G and Fig.S1B-S1C). To directly investigate the impact of stiffness mediated FOXC2 expression *in vivo*, CCl4-incuded mice liver fibrotic tissues were collected to examine FOXC2 expression. Corroborating *in vitro* findings, mice livers harvested after CCl4 injection showed prominently increased mRNA and protein expression of FOXC2 (Fig. 1H-1I and Fig.S1D), indicating FOXC2 as a mechanosensitive transcription factor that may take part in mechanotransduction of HSCs.

### 3.2 FOXC2 expression was also elevated in human liver fibrotic tissues

**Figure 2:**
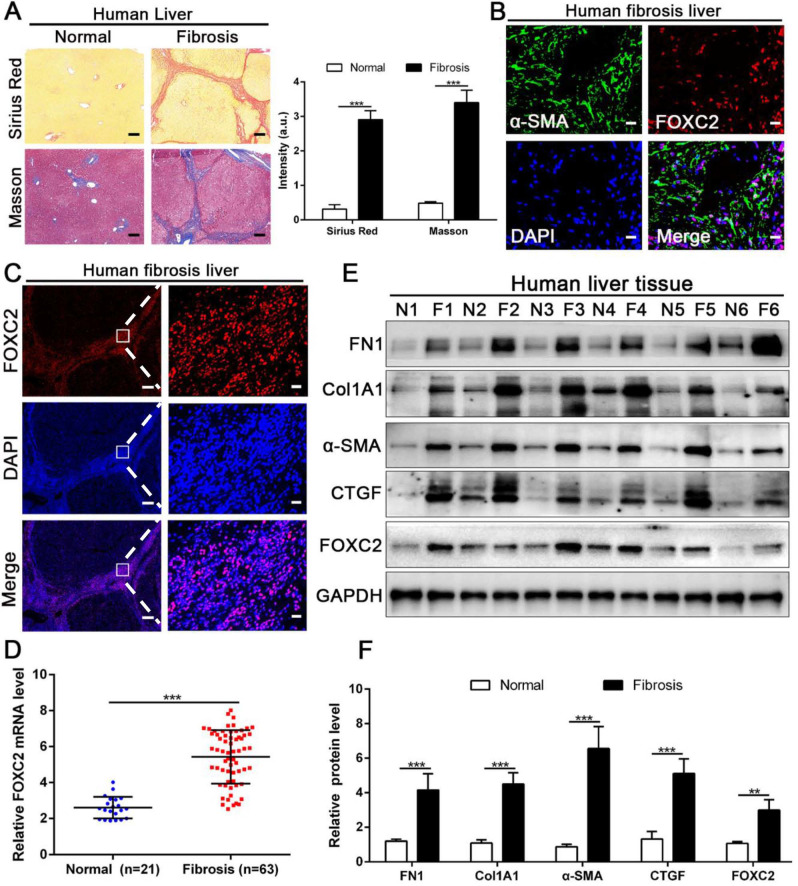
** The expression of FOXC2 was prominently increased in human fibrotic or cirrhotic liver tissues.** (A) Sirius Red and masson staining were used to confirm the liver fibrosis. Magnification is ×5, and scale bars = 200 μm. n =three independent experiments, ***P < 0.001 by Student's t-test. (B-C) IF staining showed that FOXC2 was expressed in α-SMA positive myofibroblasts and mainly located in cell nucleus. B and C right, magnification is ×40, and scale bars = 20 μm. C left, magnification is ×5, and scale bars = 200 μm. n =three independent experiments. (D-F) qRT-PCR and WB data displayed that the expression of HSCs activation markers and FOXC2 were obviously increased in human fibrotic or cirrhotic liver tissues; N = normal liver tissues and F = fibrotic or cirrhotic liver tissues. n =three independent experiments. **P<0.01, ***P<0.001 by Student's t-test.

We next examined whether FOXC2 expression in human fibrotic liver tissues. Human fibrotic or cirrhotic progression was confirmed by Sirius red and Masson staining (Fig. 2A). IF staining present that FOXC2 was expressed in α-SMA positive myofibroblasts, and mainly located in cell nucleus (Fig. 2B-2C). Prominently, fibrotic liver specimens displayed much more FOXC2 expression than normal liver samples (Fig. 2D-2F), and higher expression levels of FOXC2 were in line with an increase of fibrotic genes or myofibroblasts activation markers, such as FN1, Col1A1, CTGF and α-SMA (Fig. 2D-2F). In sum, these results indicated that FOXC2 expression was notably enhanced in fibrotic livers.

### 3.3 Depletion of FOXC2 repressed stiffness-induced HSCs activation

**Figure 3:**
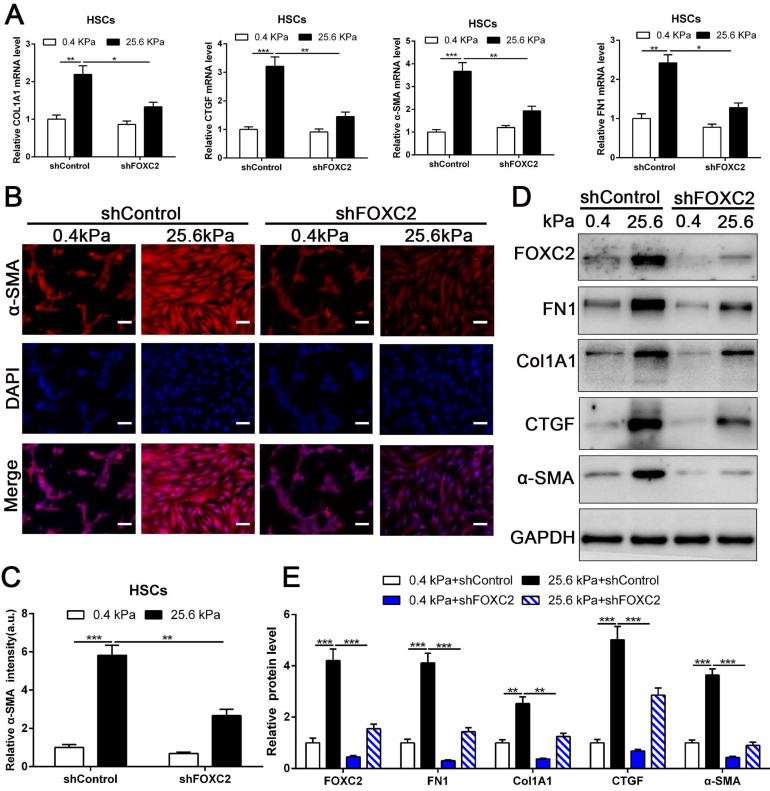
** Knockdown of FOXC2 partly offset matrix stiffness induced HSCs activation.** (A) qRT-PCR confirmed that 25.6kPa stiffness induced the upregulation of mRNA level of FN1, Col1A1, CTGF, α-SMA and FOXC2, while silencing of FOXC2 partly abrogated stiffness induced HSCs activation. n =three independent experiments, *P < 0.05, **P < 0.01, ***P < 0.001 by ANOVA. (B-C) IF staining present that 25.6kPa stiffness induced significantly upregulated α-SMA expression. Knockdown of FOXC2 repressed the 25.6kPa stiffness induced α-SMA expression. Magnification is ×10, and scale bars = 50 μm. n =three independent experiments, **P < 0.01, ***P < 0.001 by ANOVA. (D-E) WB also verified that knockdown of FOXC2 inhibited the expression level of FN1, Col1A1, CTGF and α-SMA induced by 25.6kPa stiffness. n =three independent experiments, **P < 0.01, ***P < 0.001 by ANOVA.

To directly investigate the impact of FOXC2 on stiffness-medicated HSCs activation, HSCs were infected shFOXC2 lentiviruses and then cultured on polyacrylamide hydrogels with 0.4 kPa and 25.6kPa respectively. Our qRT-PCR and WB results showed that stiffness-induced expression of α-SMA, Col1A1, FN1 and CTGF was partly abrogated by FOXC2 knockdown (Fig. 3A-3E). Additionally, IF staining showed that stiffness increased the expression of α-SMA in HSCs, while depletion of FOXC2 offset the stiffness-induced the expression of α-SMA (Fig. 3B-3C). We next used si-FOXC2 to test the role of FOXC2 in mechanotransduction of HSCs. HSCs transfected with si-RNAs encoding non-targeting siRNA (si-Control) or FOXC2 si-RNA (si-FOXC2) were seeded on 0.4 kPa or 25.6 kPa gel and collected for qRT-PCR and WB, which verified that FOXC2 knockdown repressed stiffness-mediated upregulation of FN1, Col1A1, α-SMA and CTGF in HSCs (Fig.S2A-S2B). Furthermore, ChIP assay also showed that matrix stiffness induced FOXC2 binding to the promoter of FN1, Col1A1, α-SMA and CTGF and directly upregulated the transcription of FN1, Col1A1, α-SMA and CTGF (Fig.S3A-S3D). Collectively, these results exhibited that depletion of FOXC2 repressed stiffness-induced HSCs activation into myofibroblasts.

### 3.4 Stiffness induced the upregulation of antisense lncRNA FOXC2-AS1, and shRNA-mediated FOXC2-AS1 knockdown suppressed stiffness-induced HSCs activation

**Figure 4:**
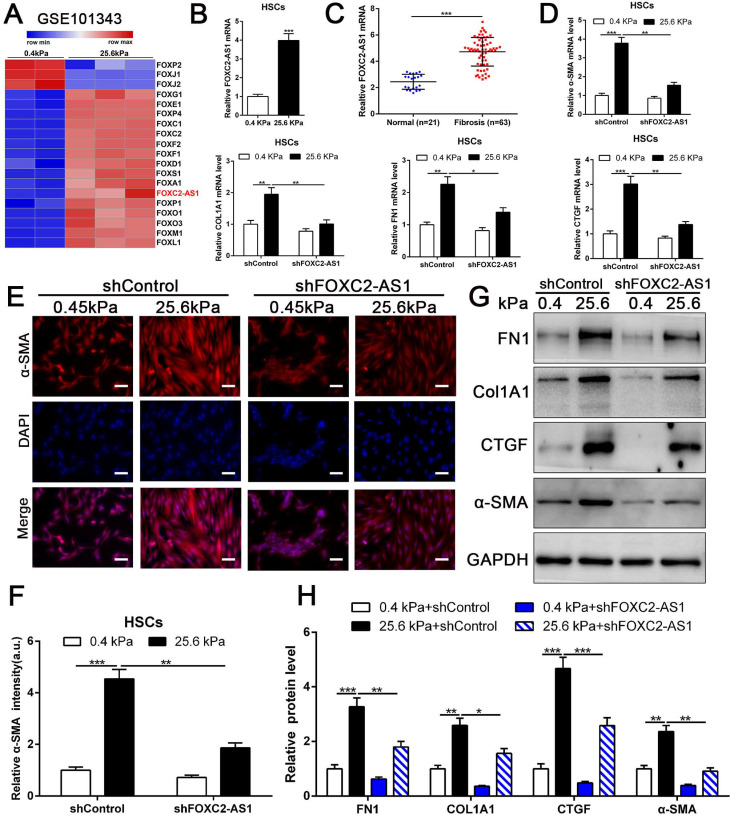
** stiffness-upregulated lncRNA FOXC-AS1 mediated matrix stiffness-induced HSCs activation.** (A) Further analysis of RNA-seq data showed that compared with 0.4kPa, 25.6kPa stiffness promoted a panel of FOX family transcription factors as well as lncRNA FOXC2-AS1. (B-C) Both *in vitro* and *in vivo* stiffness promoted the expression level of FOXC2-AS1. n =three independent experiments, ***P < 0.001 by Student's t-test. (D-H) qRT-PCR, IF and WB data displayed that lncRNA FOXC-AS1, induced by matrix stiffness, also modulated the stiffness-induced HSCs activation. Magnification is ×10, and scale bars = 50 μm. n =three independent experiments, *P<0.5, **P<0.01, ***P<0.001 by ANOVA.

Previous studies implicated FOX transcription factors, including FOXM1, FOXP1, FOXF1, FOXK1 and FOXK2^32^-^36^, exert vital roles on fibroblasts or HSCs activation. To uncover the landscape of FOX transcription factors alterations under matrix stiffness, we further analysis our RNA-seq data, and found that stiffness induced the upregulation of FOXG1, FOXE1, FOXC1/2, FOXF1/2, FOXD1, FOXS1, FOXA1, FOXP1, FOXO1/3, FOXM1, FOXL1, while downregulated FOXP2, FOXJ1/2 (Fig. 4A). Unexpectedly, the level of long non-coding RNA FOXC2-AS1 in HSCs was significantly increased under stiffness microenvironment (Fig. 4A).

Previous studies illustrated that the long non-coding RNAs (lncRNAs) exert vital roles on HSC activation^37^, ^38^. To investigate whether stiffness modulated the FOXC2-AS1 expression and mediated then HSCs activation. We seeded HSCs on 0.4 kPa and 25.6kPa hydrogels for qRT-PCR and found that the level of FOXC2-AS1 increased proportionally to stiffness *in vitro* (Fig. 4B). Next, we aspired to examine the *in vivo* expression of FOXC2-AS1 stiffness induced by stiffness. To this end, we detected the expression of FOXC2-AS1 in human fibrotic livers, and found that FOXC2-AS1 expression was obviously elevated *in vivo* (Fig 4C). These results provide *in vivo* support to our *in vitro* findings that matrix stiffness can effectively induced FOXC2-AS1 in HSCs.

However, the function of stiffness-induced FOXC2-AS1 in HSC is still unknown. We next used FOXC2-AS1 shRNA to examine the role of FOXC2-AS1 in mechanotransduction of HSCs. HSCs transduced with lentiviruses encoding FOXC2-AS1 shRNA or non-targeting shRNA (control shRNA) were then plated on 0.4 kPa or 25.6 kPa hydrogels and collected for IF, qRT-PCR and western blot, which corroborated that depletion of FOXC2-AS1 inhibited stiffness-induced upregulation of α-SMA, Col1A1, FN1 and CTGF in HSCs (Fig. 4D-4H). Thus, these data illustrated that stiffness-induced HSCs activation into myofibroblasts requires lncRNA FOXC2-AS1.

### 3.5 Stiffness-induced FOXC2-AS1 maintained the RNA stability of FOXC2

**Figure 5:**
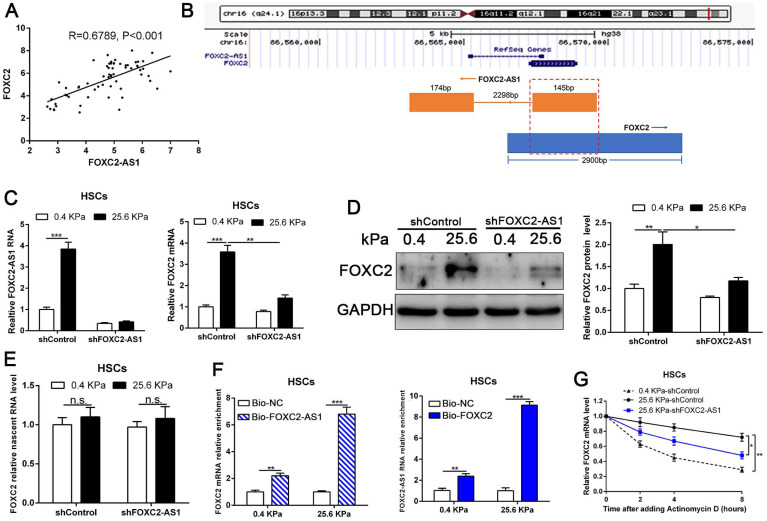
** LncRNA FOXC2-AS1, induced by stiffness, could maintain the RNA stability of FOXC2.** (A) Pearson correlation analysis revealed that there existed a positive correlation between lncRNA FOXC2-AS1 and FOXC2 in human fibrotic tissues. n = 63. (B) Schematic diagram showed that 145 bp of lncRNA FOXC2-AS1 with a full length of 319 bp overlapped with the first exon of FOXC2. (C-D) FOXC2-AS1 and FOXC2 expression level were detected by qRT-PCR and WB after FOXC2-AS1 expression was altered via gene interference. n =three independent experiments, *P<0.5, **P<0.01, ***P<0.001 by ANOVA. (E) qRT-PCR examined the level of nascent FOXC2 pre-mRNA by using Click-iT Nascent RNA Capture Kit in FOXC2-AS1-silenced and control cells. n =three independent experiments, n.s. No statistical difference. (F) RNA pull-down assay showed that biotin-labeled FOXC2-AS1 can bind with FOXC2 mRNA. Consistently, biotin-labeled FOXC2 can also interact with lncRNA FOXC2-AS1. n =three independent experiments, ***P<0.001 by ANOVA. (G) qRT-PCR was used to detect FOXC2 mRNA stability in FOXC2-AS1-depleted or control HSCs incubated with the transcriptional inhibitor actinomycin D for different times under different stiffness. n =three independent experiments, *P<0.5, **P<0.01.

Next, we interrogated the potential mechanism of functional FOXC2-AS1 mediated stiffness-induced HSCs activation. Subcellular fractionation detection and FISH array verified that FOXC2-AS1 was both located in the cytoplasm and nucleus of HSCs (Fig. S4A-S4B). A convincing body of evidence has verified that antisense lncRNAs modulated the expression level of their neighboring genes^39^. Interestingly, FOXC2-AS1 was the nearest gene, possessing some overlap sequences with FOXC2 (Fig. 5B).

Our qRT-PCR results also confirmed that statistically significant positive correlation between FOXC2-AS1 and FOXC2 expression in human fibrotic or cirrhotic livers (Fig. 5A). Moreover, previous studies showed that antisense RNA transcripts could modulate their sense genes in the transcriptional or post- transcriptional level^40^. Thus, we queried whether FOXC2-AS1 impacted the transcription or stability of FOXC2 under matrix stiffness. Depletion of FOXC2-AS1, the mRNA and protein level of FOXC2 was prominently reduced at 25.6kPa compared to control HSCs (Fig. 5C-5D). Thus, these results verified that FOXC2-AS1 positively regulated FOXC2 expression. To explore whether FOXC2-AS1 regulates FOXC2 transcription, we detected the level of nascent FOXC2 pre-mRNA using a Click-iT Nascent RNA Capture Kit in FOXC2-AS1-depleted and control HSCs on 0.4 kPa or 25.6 kPa. The results showed that depletion of FOXC2-AS1 has no influence on the level of the nascent FOXC2 pre-mRNA (Fig. 5E), which indicated that FOXC2-AS1 does not modulate FOXC2 expression at the transcriptional level. RNA pull-down results corroborated that lncRNA FOXC2-AS1 could bind with FOXC2 mRNA (Fig. 5F). Next, we explored whether FOXC2-AS1 regulated posttranscriptional process of FOXC2 mRNA by RNA-stability assay. In the presence of a transcriptional inhibitor actinomycin D, FOXC2 mRNA of HSCs on 25.6kPa degraded slower than it did on 0.4 kPa, while knockdown of FOXC2-AS1 accelerated FOXC2 degradation on 25.6 kPa (Fig. 5G), indicating that stiffness-induced FOXC2-AS1 could enhance FOXC2 mRNA stability.

### 3.6 LncRNA FOXC2-AS1 protected FOXC2 mRNA from degradation by RNA exosome complex

**Figure 6:**
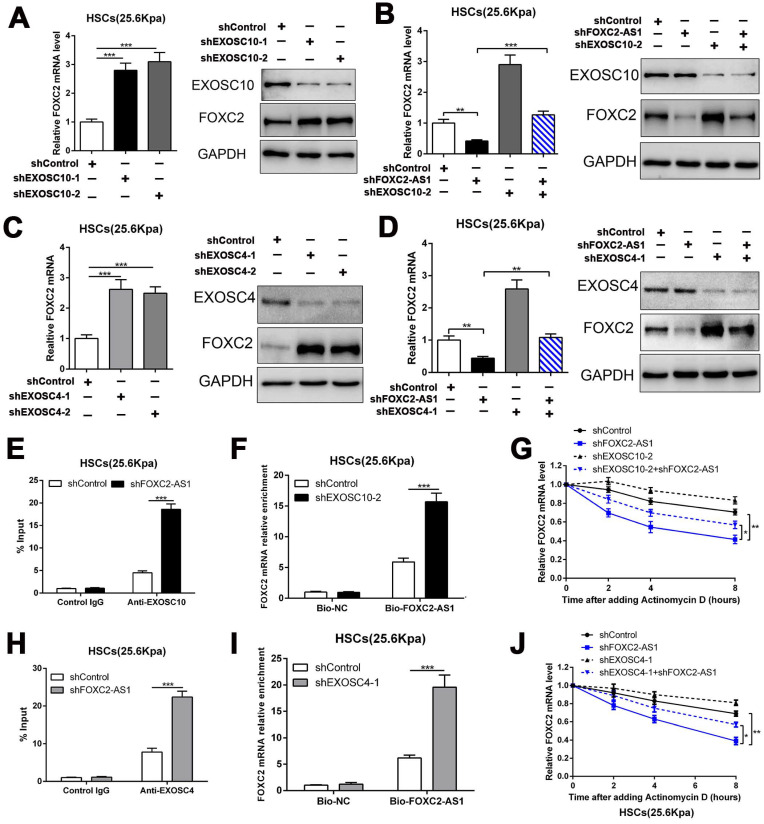
** LncRNA FOXC2-AS1 interacted with FOXC2 mRNA and protected FOXC2 mRNA from degradation by RNA exosome complex.** (A) knockdown of EXOSC10 increased the expression of FOXC2. n =three independent experiments, ***P<0.001 by ANOVA. (B) EXOSC10 depletion rescued FOXC2 mRNA and protein level in FOXC2-AS1 knockdown HSCs. n =three independent experiments, **P<0.01, ***P<0.001 by ANOVA. (C-D) Knockdown of EXOSC4, the core component of RNA exosome complex, increased the expression of FOXC2, while depletion of FOXC2-AS1 reduced the expression level of FOXC2. Simultaneously knockdown of FOXC2-AS1 and EXOSC4 in HSCs reversed the FOXC2 downregulation induced by only FOXC2-AS1 silence. n =three independent experiments, **P<0.01, ***P<0.001by ANOVA. (E) RIP assay revealed that knockdown of FOXC2-AS1 led to increased binding of FOXC2 mRNA to EXOSC10 in HSCs. n =three independent experiments, ***P<0.001 by ANOVA. (F) RNA-pull down assay demonstrated that silencing EXOSC10 resulted in elevated binding of FOXC2 mRNA to lncRNA FOXC2-AS1 in HSCs. n =three independent experiments, ***P<0.001 by ANOVA. (G) HSCs on 25.6kPa infected with the indicated shRNAs were incubated with the transcription inhibitor actinomycin D (5 mg/mL), and FOXC2 mRNA stability was assessed by qRT-PCR after collected the mRNA at the indicated time point. n =three independent experiments, *P<0.5, **P<0.01 by ANOVA. (H) RIP assay verified that knockdown of FOXC2-AS1 led to increased binding of FOXC2 mRNA to EXOSC4 in HSCs. n =three independent experiments, ***P<0.001 by ANOVA. (I) RNA-pull down assay illustrated that silencing EXOSC4 effectively increased the binding of FOXC2 mRNA to lncRNA FOXC2-AS1 in HSCs. n =three independent experiments, ***P<0.001 by ANOVA. (J) the stability of FOXC2 mRNA was evaluated in the presence of Actinomycin D. FOXC2-AS1 knockdown accelerated the degradation of FOXC2 mRNA in HSCs and this effect was partly offset by knockdown of EXOSC4. n =three independent experiments, *P<0.5, **P<0.01 by ANOVA.

To investigate how FOXC2-AS1 protects FOXC2 mRNA stability, we concentrated on the RNA exosome complex, a multiprotein complex in relation to RNA decay^22^. Exosome component 10 (EXOSC10), possessing 3'-5' exoribonuclease activity, is a catalytic component of the RNA exosome complex and also plays a crucial role on mRNA degradation^22^. Previous studies have illustrated that EXOSC10 mainly localizes to the nucleus in yeast, however, it is extensive expressed in cytoplasm and nucleus in human cells^22^. To examine whether FOXC2 mRNA is degraded by the exosome complex, HSCs infected with lentiviruses encoding EXOSC10 shRNA or non-targeting shRNA (control shRNA) were harvested for qRT-PCR, which revealed that knockdown of EXOSC10 significantly elevated the mRNA level of FOXC2 (Fig. 6A and Fig.S5A). Additionally, the FOXC2 mRNA level, decreased by depletion of FOXC2-AS1 in HSCs, was reversed by EXOSC10 knockdown (Fig. 6B and Fig. S5B). To test whether FOXC2-AS1 protects FOXC2 mRNA stability by competing of EXOSC10/FOXC2 mRNA binding, FOXC2-AS1 knockdown HSCs seeded on 25.6 kPa were harvested for RIP. Our RIP data illustrated that FOXC2-AS1 knockdown resulted in elevated EXOSC10/FOXC2 mRNA binding in HSCs (Fig. 6E). Consistently, RNA pull-down assay also verified that the depletion of EXOSC10 induced a remarkable increase binding between lncRNA FOXC2-AS1 and FOXC2 mRNA (Fig. 6F). RNA stability assay verified that depletion of FOXC2-AS1 accelerated the degradation of FOXC2 mRNA in HSCs and that this alteration was offset by knockdown of both FOXC2-AS1 and EXOSC10 (Fig. 6G). Therefore, the RNA exosome complex is responsible for FOXC2 mRNA decay in HSCs.

To further elucidate the efficacy of the RNA exosome in the RNA stabilizing function of FOXC2-AS1, we further assessed the role of EXOSC4, another crucial component of the core of the RNA exosome, on FOXC2 mRNA stability. Like EXOSC10, silencing EXOSC4 indeed increased the mRNA and protein level of FOXC2 (Fig. 6C and Fig S5C), moreover, knockdown of FOXC2-AS1 induced the downregulation of FOXC2 mRNA was partly reversed by EXOSC4 silence (Fig. 6D and Fig.S5D). The RIP assay validated that EXOSC4 interacted with FOXC2 mRNA, and that silencing of FOXC2-AS1 could elevate the binding affinity of EXOSC4 with FOXC2 mRNA (Fig. 6H). Meanwhile, RNA pull-down results also demonstrated that depletion of EXOSC4 greatly enhanced the binding of lncRNA FOXC2-AS1 with FOXC2 mRNA (Fig. 6I). Knockdown of FOXC2-AS1 induced the degradation of FOXC2 mRNA could partly reversed by EXOSC4 silence (Fig. 6J). Cumulatively, these data showed that stiffness-induced lncRNA FOXC2-AS1 maintained the RNA stability of FOXC2, and protected FOXC2 mRNA from degradation by RNA exosome complex.

### 3.7 Stiffness-induced FOXC2-AS1 medicated HSCs activation into myofibroblasts depend on FOXC2

**Figure 7:**
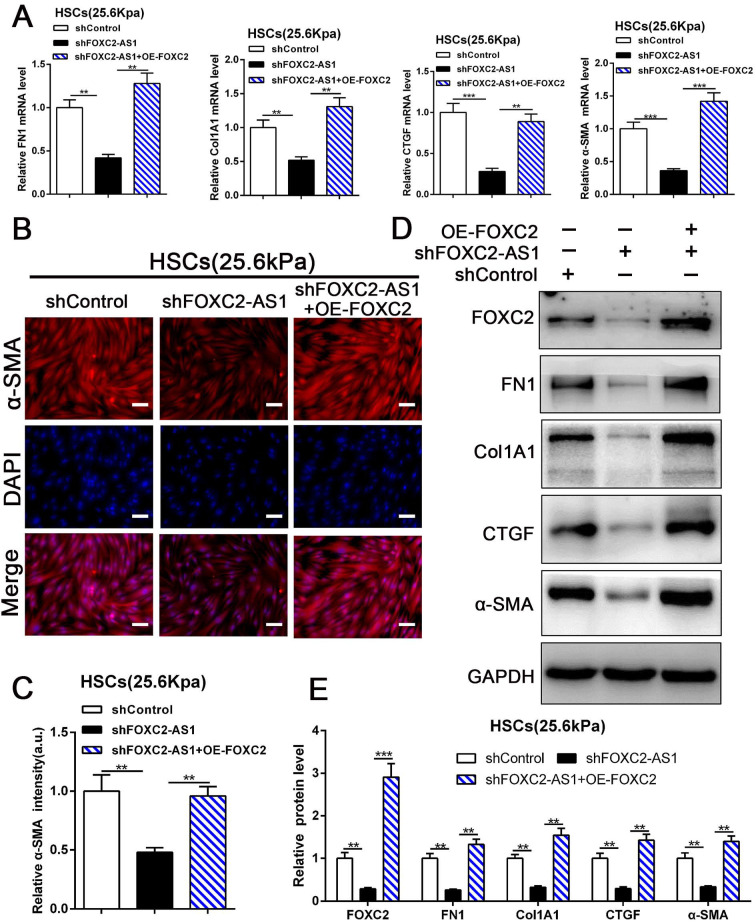
** FOXC2-AS1/FOXC2 axis mediated stiffness-induced HSCs activation.** (A) Knockdown of FOXC2-AS1in HSCs plated on 25.6kPa inhibited the mRNA level of FN1, Col1A1, CTGF and α-SMA, while overexpression of FOXC2 effectively rescued the inhibition of HSCs activation induced by FOXC2-AS1 knockdown. n =three independent experiments, **P<0.01, ***P<0.001 by ANOVA. (B-E) IF and WB data displayed that overexpression of FOXC2 could reverse the FOXC2-AS1 knockdown induced the repression of HSCs activation. Magnification is ×10, and scale bars = 50 μm. n =three independent experiments, **P<0.01, ***P<0.001 by ANOVA.

Next, we aspired to investigate FOXC2-AS1/FOXC2 axis mediated HSCs activation under matrix stiffness microenvironment. Therefore, we performed rescue assays to evaluate whether FOXC2-AS1 mediated stiffness-induced HSCs activation *via* FOXC2. As shown in Fig. 7A-7E, knockdown of FOXC2-AS1 partly suppressed stiffness-induced HSCs activation, while ectopic expression of FOXC2 could notably rescue the depletion of FOXC2-AS1 mediated inhibition of HSCs activation, as determined by IF, qRT-PCR and western blot. Thus, FOXC2-AS1 mediated stiffness-induced HSCs activation *via* FOXC2.

### 3.8 Targeting FOXC2 alleviated CCL4 or BDL-induced mice liver fibrosis *in vivo*

**Figure 8:**
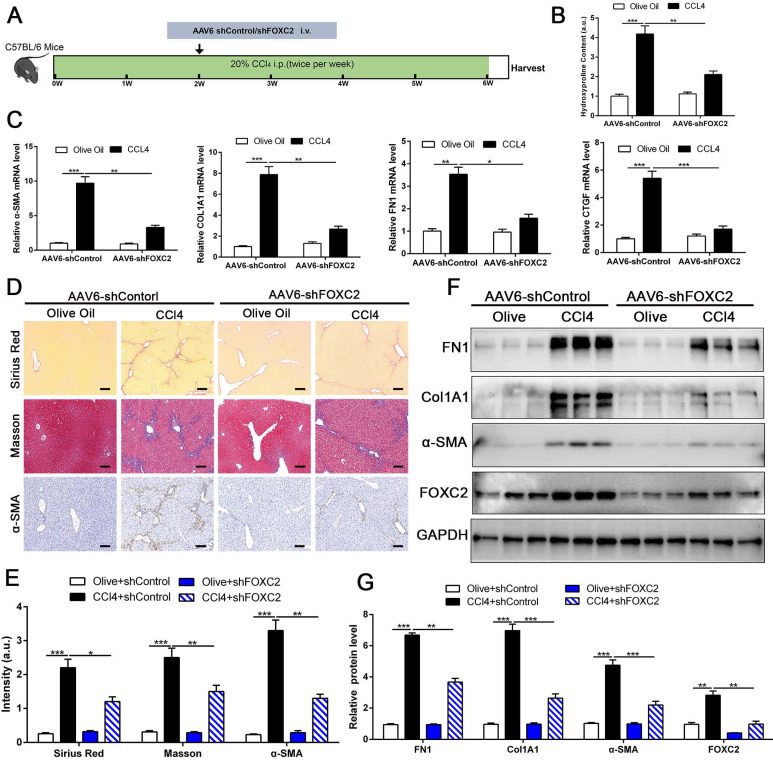
** Depletion of FOXC2 alleviated CCl4-induced mouse liver fibrosis.** (A) Schematic overview of the experimental design. (B) Collagen I content was reduced in livers of FOXC2 knockdown compared to matched WT controls induced by CCl4, as assayed by hydroxyproline assay. n =6, **P<0.01, ***P<0.001 by ANOVA. (C) The mRNA was harvested from whole liver and qRT-PCR was conducted to analyze FN1, Col1A1, α-SMA and CTGF expression. (D-E) Sirius Red, masson staining and IHC were performed to assess liver fibrosis and α-SMA expression. Magnification is ×10, and scale bars = 100 μm. n =6, *P<0.5, **P<0.01, ***P<0.001 by ANOVA. (F-G) WB showed that CCl4-induced the expression of FN1, Col1A1, α-SMA, CTGF and FOXC2, while knockdown of FOXC2 *in vivo* by *AAV6* shFOXC2 effectively impeded FN1, Col1A1, α-SMA and CTGF expression. n =6, **P<0.01, ***P<0.001 by ANOVA.

To investigate whether targeting FOXC2 could repress further progression of established liver fibrosis *in vivo*, we utilized AAV6 carrying shRNA targeting FOXC2 to intervene CCl4 or BDL-induced mice fibrosis model. Increasing evidence revealed that AAV6 displayed organ tropism for activated HSCs or myofibroblasts in the liver during fibrosis^28^, and the targeting efficacy of AAV6 is reach to 17.04 % in nestin^+^ positive murine activated HSCs 28. Following this, we utilized AAV6-shFOXC2 in liver fibrotic mice to assess the function of FOXC2 in CCl4 or BDL-induced liver fibrosis *in vivo*. The administration of AAV6-shControl barely impacted the liver histology, liver weight, body weight and liver function of the mice (Fig. S6A-S6D). We isolated the activated HSCs in mouse liver from CCl4 model after tail vein injection of AAV6 FOXC2 shRNA, then used qRT-PCR and western blot to detect the level of FOXC2. Our results confirmed that the expression of FOXC2 was decreased in mouse activated HSCs after tail vein injection of *AAV6* FOXC2 shRNA (Fig. S7A-S7B), which were consistent with previous results^27^, ^28^.Furthermore, the Sirius Red staining, masson staining and hydroxyproline analysis exhibited that CCl4 injection or BDL induced mice liver fibrosis, while the degree of fibrogenesis was alleviated upon AAV6-shFOXC2 treatment (Fig. 8A-8B, Fig.8D-8E, Fig.S8A-S8C and Fig.S9B). Further analysis showed that the expression of FOXC2, α-SMA, Col1A1, FN1and CTGF, and hepatocellular damage were also reduced following with *AAV6*-shFOXC2 treatment in CCl4 or BDL-induced liver fibrosis model (Fig. 8C, Fig. 8F-8G, Fig S6E, Fig. S9A, Fig. S9C-9D). Additionally, IHC staining of CK-19 in BDL model also showed that the cholangiocytes proliferated area was also decreased after targeting FOXC2 by AAV6-shRNA *in vivo* (Fig. S10A-B). Totally, these data showed that targeting FOXC2 by using AAV6 alleviated liver fibrosis *in vivo*.

## 4. Discussion

A convincing body of evidence has put forth that matrix stiffness is a defining feature of liver fibrosis, and also recognized to contribute profoundly to disease progression *via* mechano-activation of myofibroblasts originated from HSCs^9^. Therefore, efforts have been ongoing to investigate the mechanistic alterations underlying stiffness-mediated HSCs activation and hepatic fibrosis. External matrix stiffness is relayed into intracellular signaling cascades that eventually converge onto transcriptional factors^10^. Now, some of the key-modulators of stiffness have already been identified, such as YAP, TAZ, SRF and ZNF416, whose target genes are frequently overexpressed in pathological fibrosis tissue^10^-^12^. Here, through analysis the RNA-seq data of primary HSCs cultured on 0.4 kPa and 25.6 kPa hydrogel, we discovered FOXC2 as a new mechanosensing transcriptional regulator that coordinate HSCs responses to the matrix stiffness. We found that matrix stiffness induces HSCs activation into myofibroblasts and enhanced nuclear accumulation of FOXC2, moreover, FOXC2 was highly expressed in fibrosis or cirrhosis tissues. Knockdown of FOXC2 impeded stiffness-induced expression of α-SMA, Col1A1, FN1 and CTGF, the makers of HSCs activation into myofibroblasts. Thus, based on the important functional roles verified here for FOXC2, further elucidation of its roles in the upstream gap linking matrix stiffness to its transcriptional functions is likely to present additional insights relevant to hepatic fibrosis or wound healing.

Previous studies have revealed that antisense lncRNAs could modulate the expression of their neighboring genes^39^. Intriguingly, antisense lncRNA FOXC2-AS1 was the nearest gene, possessing some overlap sequences with FOXC2. Further mechanistic studies displayed that stiffness was able to upregulate lncRNA FOXC2-AS1 expression, and stiffness-upregulated lncRNA FOXC2-AS1 had no influence on transcription of FOXC2. However, FOXC2-AS1 exerted its biological function through maintaining the RNA stability of FOXC2, and protecting FOXC2 mRNA from degradation by RNA exosome complex. Additionally, rescue assays confirmed that manipulation of FOXC2-AS1/FXOC2 axis effectively disrupted the matrix stiffness-mediated HSCs activation into myofibroblasts. Taken together, our results corroborate FOXC2-AS1/FOXC2 axis was identified as the key regulators of HSCs mechanoactivation and fibrogenic function.

**Figure 9:**
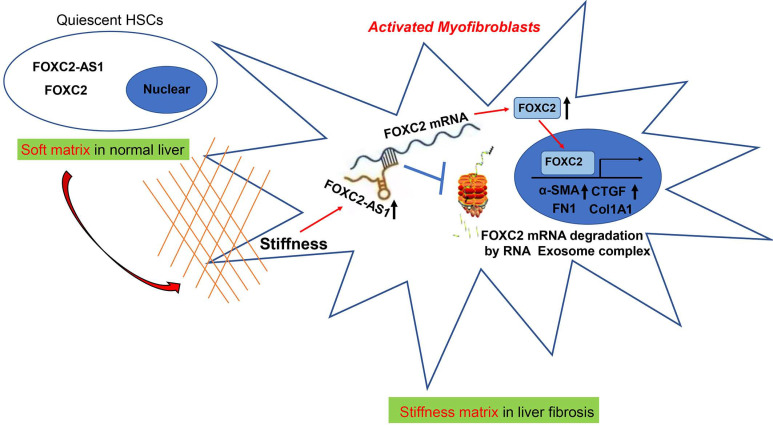
Schematic findings of the current study.

In eukaryotic cells, the RNA exosome core possesses a barrel-like structure (constituted by EXOSC4, EXOSC5, EXOSC6, EXOSC7, EXOSC8, and EXOSC9) and a cap (constituted by EXOSC1, EXOSC2, and EXOSC3)^41^, moreover, EXOSC10 has the 3'-5' exoribonuclease activity and is a crucial catalytic component of the RNA exosome. The RNA exosome complex is a multiprotein complex that exerts a profound role in modulating RNA turnover^41^-^44^. However, the role of the RNA exosome complex in regulating RNA turnover to impact HSCs activation under stiffness microenvironment is still unclear. Long non-coding RNAs (lncRNAs) are functional RNA transcript longer than 200 nucleotides, which are regarded as a redundant transcriptional product^45^. According to the features of their genomic locations, lncRNAs are sorted as sense, antisense, intergenic, intronic, or bidirectional. Some lncRNAs are exclusively localized in the cytoplasm or nucleus, while others are identified in both^45^. LncRNAs could act as decoys, signals, guides, and scaffolds by binding with protein, RNA or DNA molecules. Especially, Antisense RNA transcripts could modulate their sense genes at the transcriptional or post- transcriptional level^40^. In consistent with previous studies^19^, we found that the antisense lncRNA FOXC2-AS1 induced by stiffness maintained the mRNA stability of FOXC2, the sense gene closed to FOXC2-AS1, and protects FOXC2 mRNA degradation by RNA exosome complex in HSCs. The schematic findings of the current study were displayed in Figure 9. Hence, the findings of the correlation between the lncRNA FOXC2-AS1 and RNA exosome to maintain FOXC2 mRNA balance in the context of stiffness microenvironment indicates that the RNA exosome may also be considered as a novel therapeutic target for reprograming HSCs activation and fibrosis disease.

Recently, the study reported that an *AAV6* vector carrying transcription regulators was able to successfully reprogram activated hepatic stellate cells into hepatocytes and thus contribute to mouse hepatic fibrogenesis recovery^28^. Chen *et al*. uncovered that *AAV6* displayed organ tropism for activated HSCs or myofibroblasts in the liver during fibrosis, and the targeting efficacy of *AAV6* was reach to 17.04 % in nestin^+^ murine activated HSCs of CCl4-induced liver fibrosis model^27^. Most importantly,* AAV* vectors have now been considered as a safe and efficient gene delivery tool in clinical trials since they possess low oncogenicity and weak immunogenicity^46^, ^47^. Hence, we utilized *AAV6* vectors carrying shFOXC2 to target activated HSCs and investigate the efficacy of *AAV6* shRNA. Our *in vitro* data present the evidence of the critical involvement of FOXC2-AS1/FOXC2 signaling in stiffness mediated HSCs activation. Consistently, *in vivo* analyses of targeting FOXC2 by *AAV6* shRNA in CCl4 or BDL-induced liver fibrosis model substantiate our *in vitro* findings. We provided evidence to exhibit that applying with an* AAV6* shRNA to effectively impair FOXC2 expression in activated HSCs could mitigate hepatic fibrogenesis in mouse fibrosis model, providing a promising anti-fibrotic strategy. Moreover, besides *AAV* vectors, liposomes, EV or other new nanomaterial-based targeted delivery tools have been identified in recent years. These vectors have showed better targeting efficiency and great promise for treatment of hepatic fibrosis^48^-^50^. Hence, it is of great interest to further investigate other therapeutic approaches by targeting FOXC2 or FOXC2-AS1, which may provide novel insights in the exploitation of anti-fibrosis strategies. In sum, our study uncovers a reciprocal crosstalk between matrix stiffness and FOXC2-AS1/FOXC2 axis leading to modulation of HSCs mechanoactivation and liver fibrosis, and present *AAV6* shRNA as an effective strategy that targets FOXC2 leading to liver fibrosis resolution.

## Supplementary Material

Supplementary figures and tables.

## Abbreviations

ALD: alcoholic liver disease
NASH: non-alcoholic steatohepatitis
ECM: extracellular matrix
HSCs: hepatic stellate cells
TGF-β: transforming growth factor beta
FOXC2: forkhead box protein C2
EMT: epithelial-mesenchymal transition
BDL: bile duct ligation
LncRNAs: long non-coding RNAs
