# Supplementary material for: FOXC2-AS1/FOXC2 axis mediates matrix stiffness-induced trans-differentiation of hepatic stellate cells into fibrosis-promoting myofibroblasts
Source: Int J Biol Sci. 2023 Aug 6;19(13):4206–22. doi: 10.7150/ijbs.81581 (PMC10496501; doi:10.7150/ijbs.81581)
Supplement: Supplementary file 1 — Supplementary figures and tables. [file ijbsv19p4206s1.pdf]

Supplementary Table 1 Primers sequences for real-time PCR analysis

Gene	Primer Sequences
hFOXC2	Forward: 5′-ATCACCTTGAACGGCATCTAC-3′<br>Reverse: 5′-TTGACGAAGCACTCGTTGAG-3′
hFOXC2-AS1	Forward: 5′-CCTTCCTGGCTGTTCATCGG-3′<br>Reverse: 5′-TGGAAATAAGTGGCACGCCC-3′
hFN1	Forward: 5′-CCACAGTGGAGTATGTGGTTAG-3′<br>Reverse: 5′-CAGTCCTTTAGGGCGATCAAT-3′
hCol1A1	Forward: 5′-CAGACTGGCAACCTCAAGAA-3′<br>Reverse: 5′-CAGTGACGCTGTAGGTGAAG-3′
hα-SMA	Forward: 5′-GATGGTGGGAATGGGACAAA-3′<br>Reverse: 5′-GCCATGTTCTATCGGGTACTTC-3′
hCTGF	Forward: 5′-GCTGACCTGGAAGAGAACATTA-3′<br>Reverse: 5′-TGCAGCCAGAAAGCTCAA-3′
hGAPDH	Forward: 5′-ACATCGCTCAGACACCATG-3′<br>Reverse: 5′-TGTAGTTGAGGTCAATGAAGGG-3′
mFOXC2	Forward: 5′-GATCACTCTGAACGGCATCTAC-3′<br>Reverse: 5′-CACTTTCACGAAGCACTCATTG-3′
mFN1	Forward: 5′-TCCTGTCTACCTCACAGACTAC-3′<br>Reverse: 5′-CACTTTCACGAAGCACTCATTG-3′
mCol1A1	Forward: 5′-GAGCGGAGAGTACTGGATCG-3′<br>Reverse: 5′-GCTTCTTTTCCTTGGGGTTC-3′
mα-SMA	Forward: 5′-AAACAGGAATACGACGAAG-3′<br>Reverse: 5′-GAATGATTTGGAAAGGA-3′
mCTGF	Forward: 5′-ACTATGATGCGAGCCAACTG-3′<br>Reverse: 5′-CTCCAGTCTGCAGAAGGTATTG-3′
mGAPDH	Forward: 5′-AACAGCAACTCCCACTCTTC-3′<br>Reverse: 5′-CCTGTTGCTGTAGCCGTATT-3′

Supplementary Table 2 Primers sequences for ChIP-qPCR analysis

Gene	Primer Sequences
hFN1	Forward: 5′-TGGGTCACAAAGATTCCTCAA-3′<br>Reverse: 5′-GCCTGACACATTAAAGACACAAA-3′
hCol1A1	Forward: 5′-CTGGGAAGGAGGGTCTCTAA-3′<br>Reverse: 5′-TAGTGGGAGGCCTGTGAT-3′
hα-SMA	Forward: 5′-GGAGAAATGGAAGATAAACTGCAAA-3′<br>Reverse: 5′-GCAGATGAGTCAAATACAAAGATCAG-3′
hCTGF	Forward: 5′-CGATCTTTGCACCACAAACAG-3′<br>Reverse: 5′-GCCGCATCATTTGACATTGG-3′

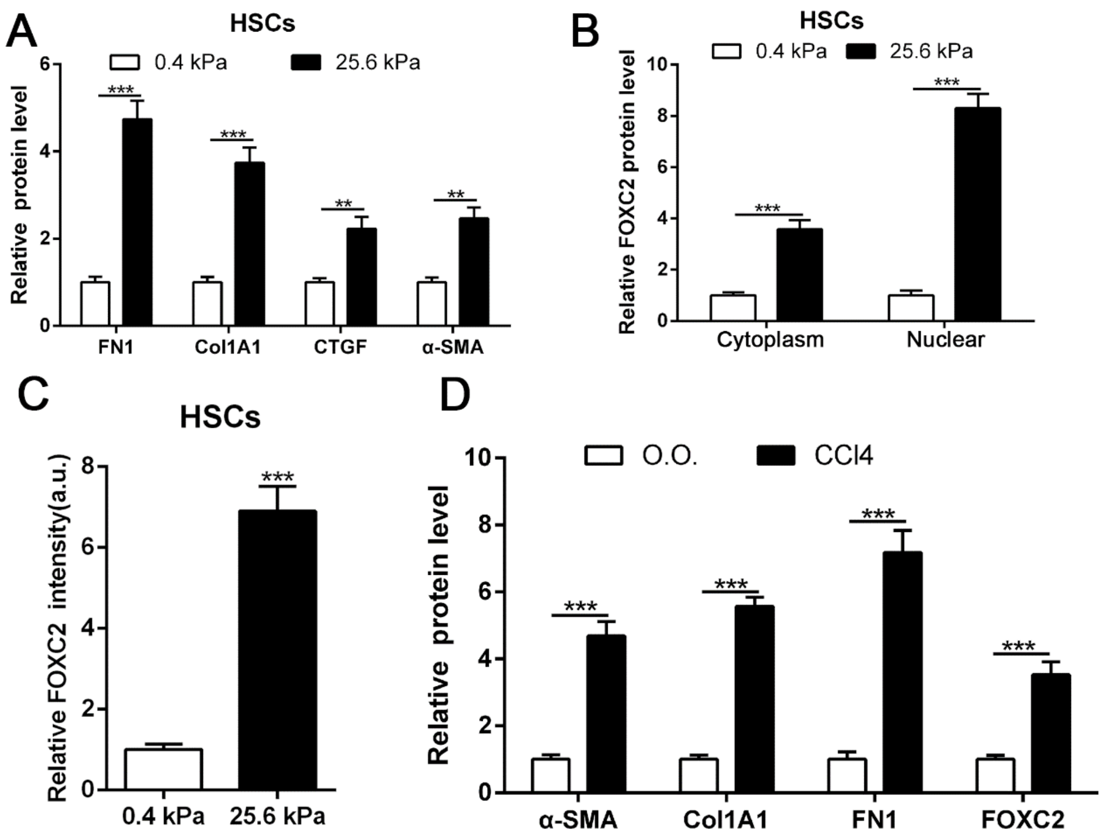

Figure S1 Relative expression of HSCs activation markers and FOXC2 induced by matrix stiffness. (A-B) Relative protein level of HSCs activation markers and FOXC2 after plated on 0.4 or 25.6kPa hydrogels. n =three independent experiments, ***P*<0.01, ****P*<0.001 by Student's t-test. (C) IF intensity alterations of FOXC2 after cultured on 0.4 or 25.6kPa hydrogels. n =three independent experiments, ****P*<0.001 by Student's t-test. (D) Relative protein level of HSCs activation markers and FOXC2 in CCl4-indcued fibrotic mice livers. n =three independent experiments, ****P*<0.001 by Student's t-test. O.O. (olive oil).

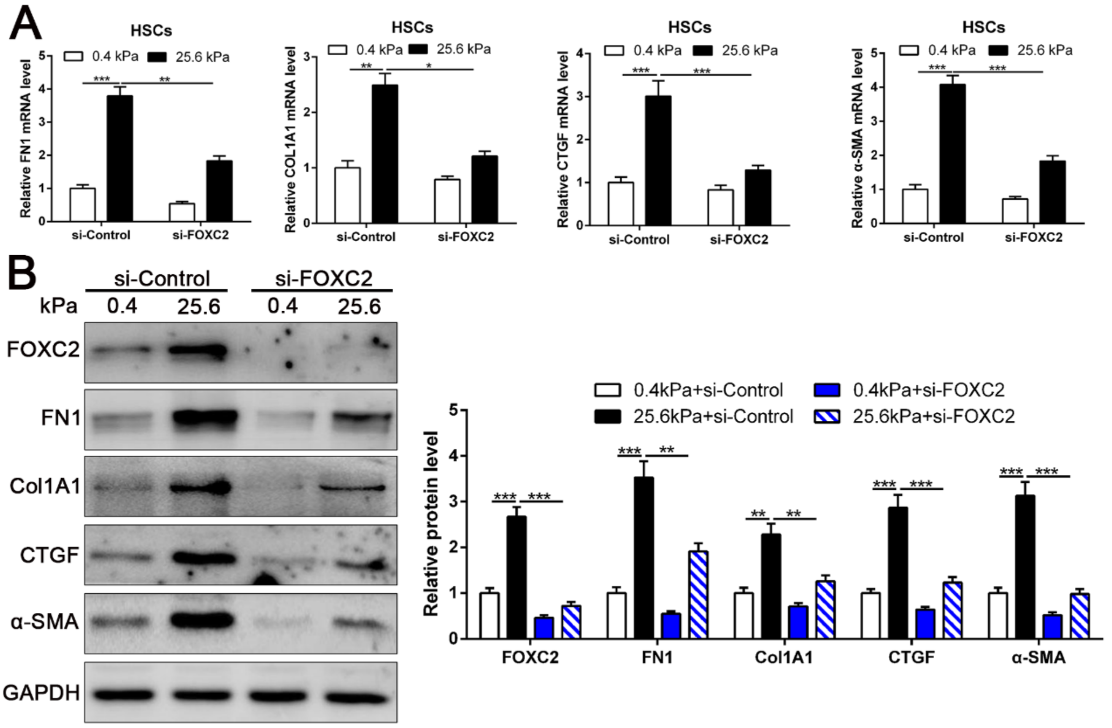

Figure S2 The FOXC2 is dispensable for matrix stiffness-induced HSCs activation. qRT-PCR (A) and western blot (B) displayed that stiffness induced the upregulation of HSCs activation markers, while targeting FOXC2 by si-RNA partly abrogated the stiffness induced HSCs activation. n =three independent experiments, **P*<0.5, ***P*<0.01, ****P*<0.001 by ANOVA.

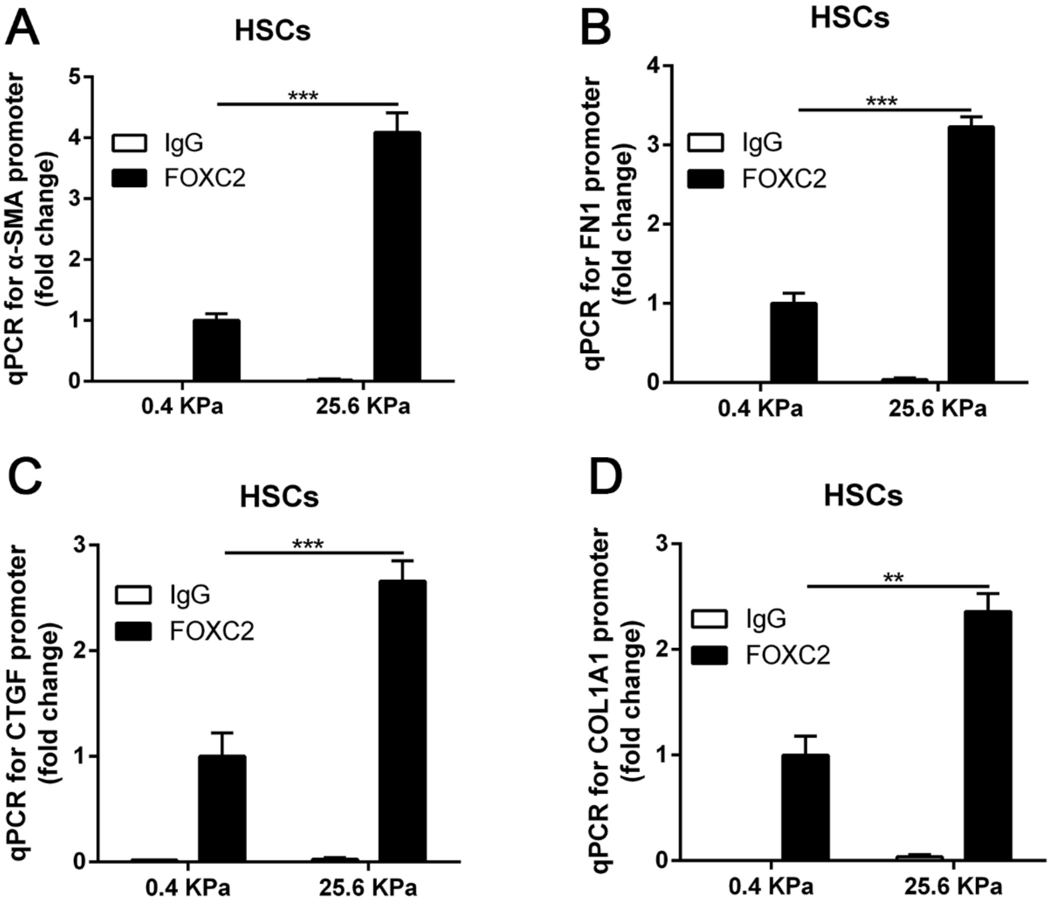

Figure S3 Matrix stiffness directly facilitated the transcription of HSCs activation markers by FOXC2. (A-D) FOXC2/DNA complexes were pulled down by control IgG or anti-FOXC2 for ChIP assay, and qPCR was performed for evaluation of FOXC2 binding to the promoter of FN1, α-SMA, CTGF and Col1A1. Stiffness increased the recruiting of FOXC2 to the promoters of FN1, α-SMA, CTGF and Col1A1 and elevated their transcription. n =three independent experiments, ***P*<0.01, ****P*<0.001 by ANOVA.

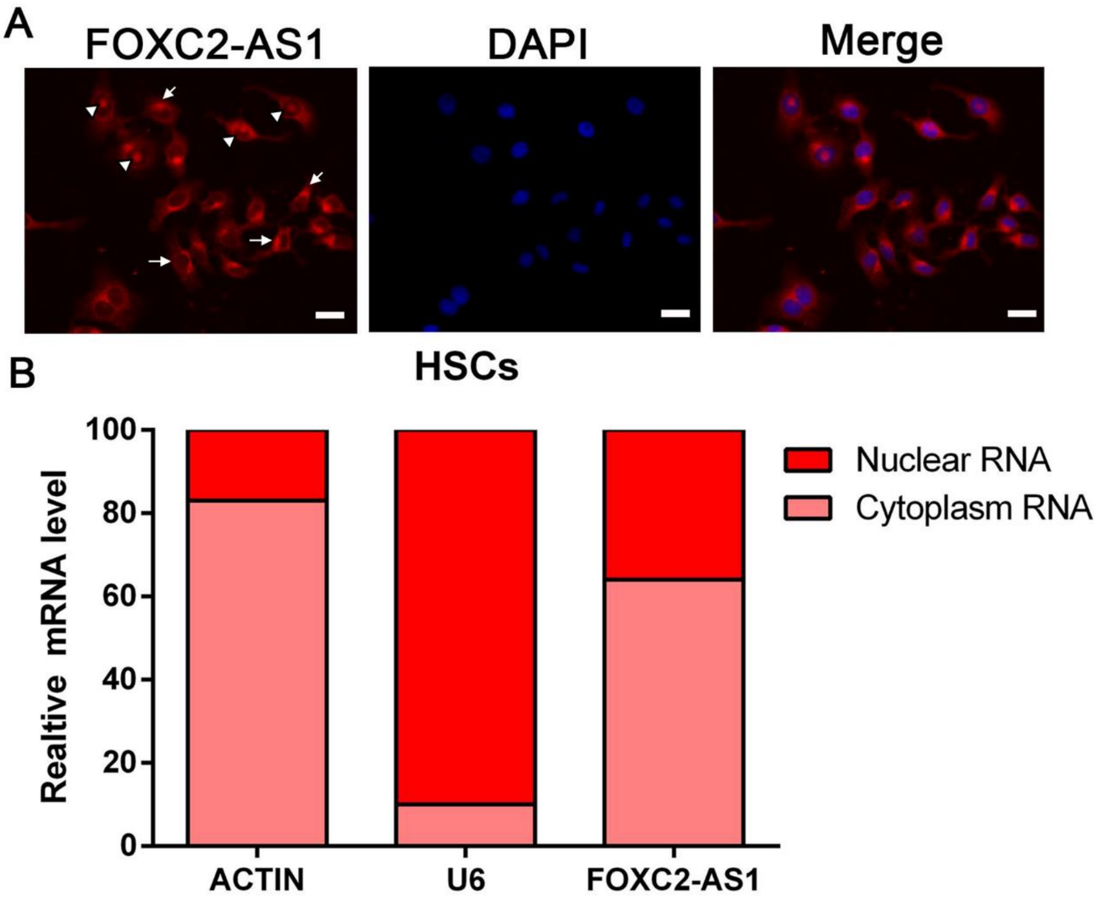

Figure S4 LncRNA FOXC2-AS1 is abundant both in cytoplasm and nucleus. (A) FISH assay and subcellular fractionation assay (B) validated that FOXC2-AS1 was located both in cytoplasm and nucleus. Magnification is ×40, and scale bars = 20 μm.

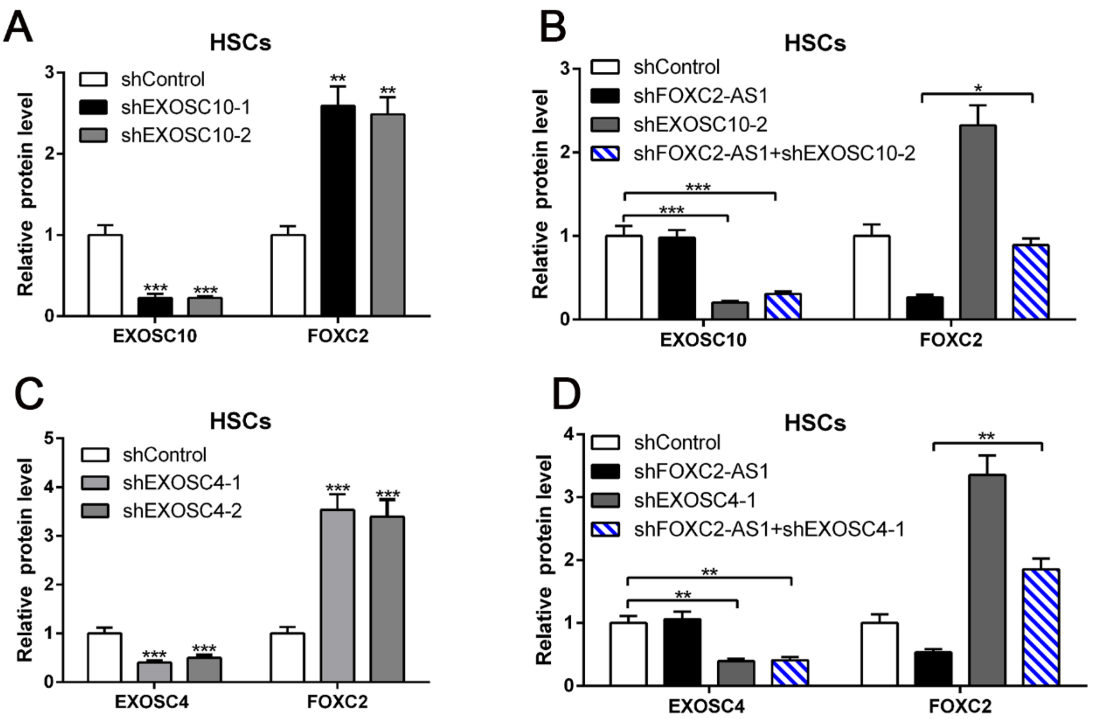

Figure S5 Relative protein level of FOXC2 after manipulation expression of lncRNA FOXC2-AS1 or core components of RNA exosome complex in HSCs. Knockdown of EXOSC10 (A) or EXOSC4 (C) increased FOXC2 level of HSCs plated on 25.6 kPa. Depletion of FOXC2-AS1(B, D) repressed FOXC2 expression, while simultaneously knockdown of FOXC2-AS1 and EXOSC10 (B) or EXOSC4 (D) partly reversed only FOXC2-AS1 silencing induced the inhibition of FOXC2. n =three independent experiments, **P*<0.5, ***P*<0.01, ****P*<0.001 by ANOVA.

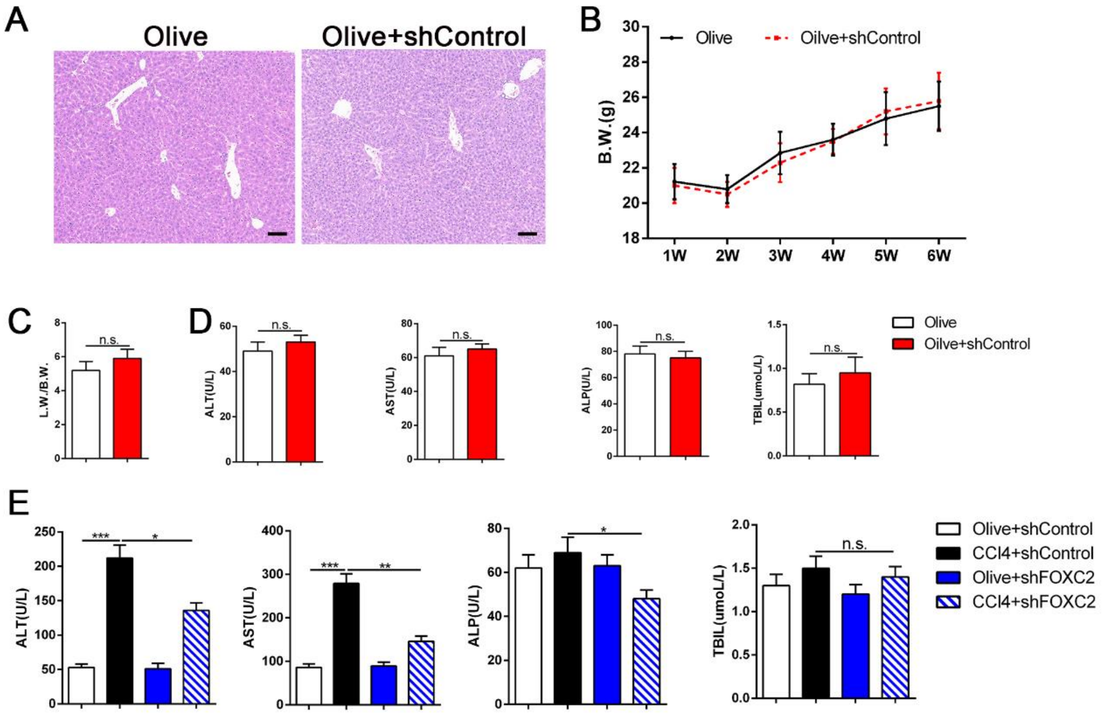

Figure S6 ***AAV6*** **therapy in mouse CCl4-induced liver fibrosis model**. (A) Representative images of HE staining in oil and oil + AAV6-shControl group. Magnification is ×10, and scale bars = 100 μm. (B) Body weight and (C) the ratio of liver weight/body weight in indicated groups. n= 6/group. n.s. no statistical difference. (D) Levels of ALT, AST, ALP, and TBIL in serum from *C57BL/6* mice in Olive or Olive+shControl group. n= 6/group. n.s. no statistical difference. (E) Levels of ALT, AST, ALP and TBIL in serum from *C57BL/6* mice in indicated groups at the time of sacrifice. n = 6/group. n.s. no significant statistical difference, **P*<0.5, ***P*<0.01, ****P*<0.001 by ANOVA.

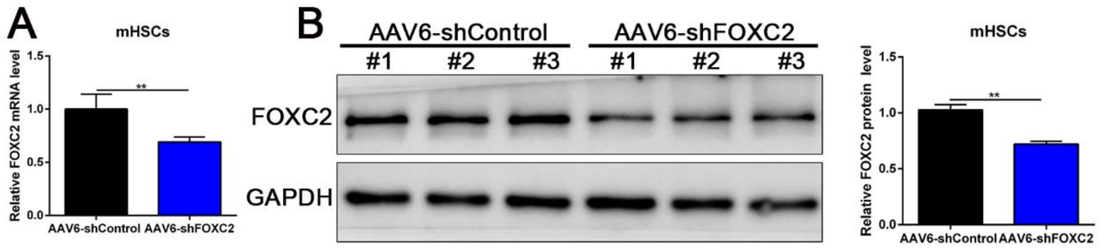

Figure S7 The activated hepatic stellate cells in mouse liver are targeted by *AAV6*

shFOXC2. The activated hepatic stellate cells in CCl4 model after tail vein injection of *AAV6* FOXC2 shRNA were isolated, and then qRT-PCR (A) and western blot (B) were used to detect the targeting efficacy of FOXC2 by *AAV6* shRNA. n = 6/group, (B left) just showed the representative image of western blot with 3 mice, $**P < 0.01$ by t-test.

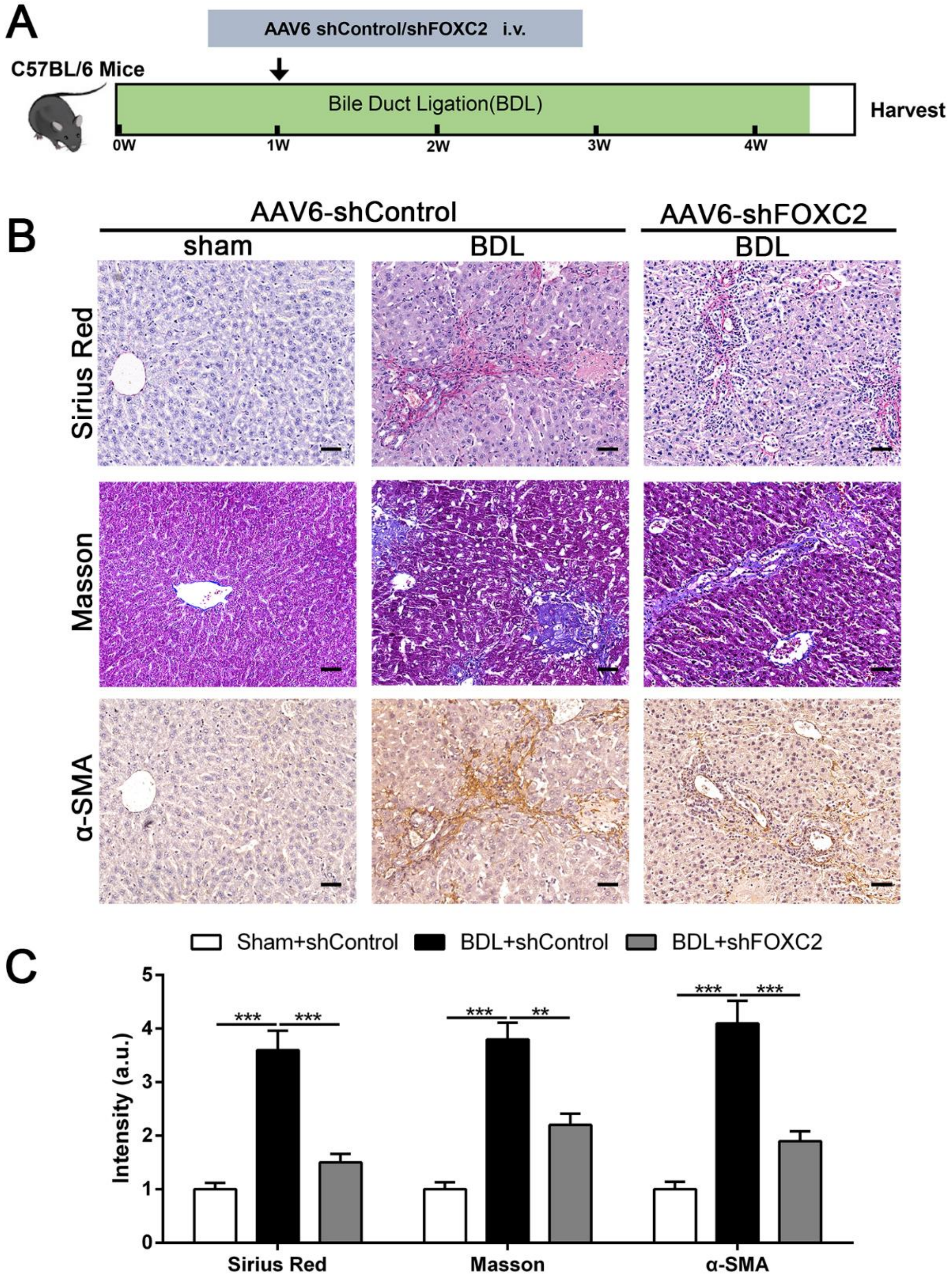

Figure S8 Reduction of FOXC2 by *AAV6* therapy resulted in reduced fibrosis in response to BDL. (A) Schematic overview of the experimental design of BDL mice fibrosis model. (B) Representative Sirius Red staining, masson staining and IHC for α-SMA. Magnification is ×10, and scale bars = 100 μm. (C) Quantification for Sirius Red staining, masson staining and α-SMA in liver tissues of indicated groups. n = 6/group. ***P*<0.01, ****P*<0.001 by ANOVA.

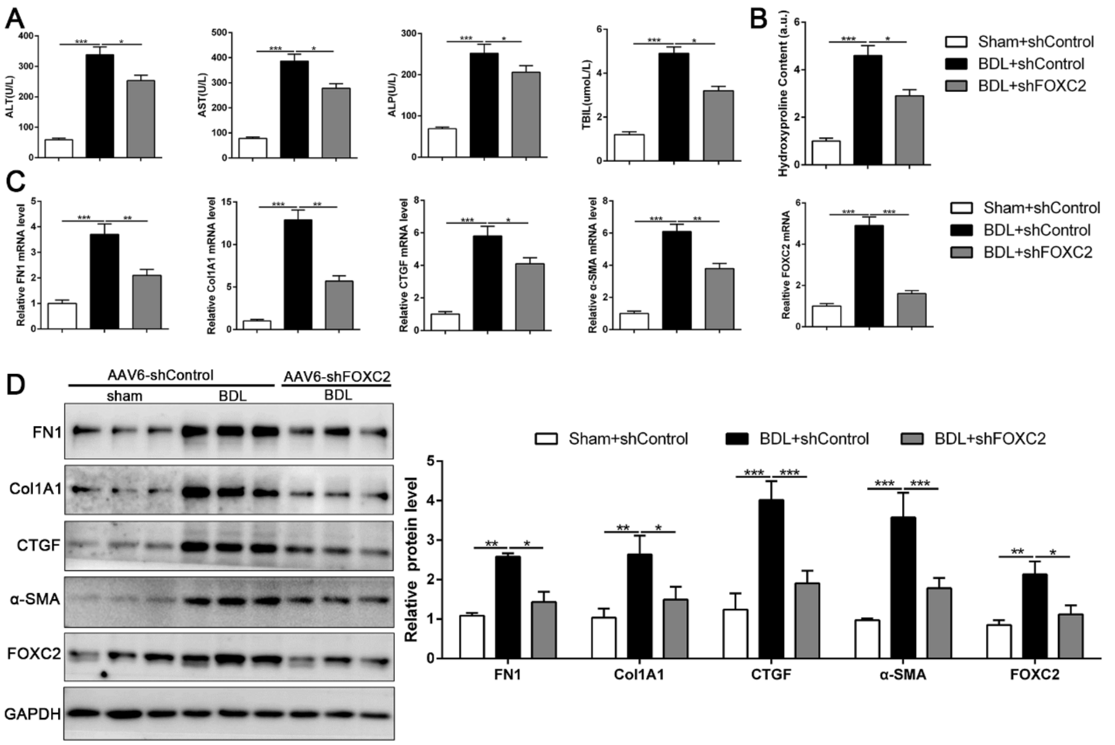

Figure S9 ***AAV6-targeted*** **FOXC2** ***in vivo*** **inhibited BDL-induced mice liver fibrosis**. (A) Levels of ALT, AST, ALP and TBIL in serum from *C57BL/6* mice in different groups at the time of sacrifice. n = 6/group. **P*<0.5, ***P*<0.01, ****P*<0.001 by ANOVA. (B) Collagen I content within harvested mice livers was detected through a Hydroxyproline assay. n = 6/group. **P*<0.5, ****P*<0.001 by ANOVA. (C-D) qRT-PCR and WB were used to test the HSCs activation markers (FN1, Col1A1, CTGF, α-SMA) and FOXC2 alterations. n = 6/group. **P*<0.5, ***P*<0.01, ****P*<0.001 by ANOVA.

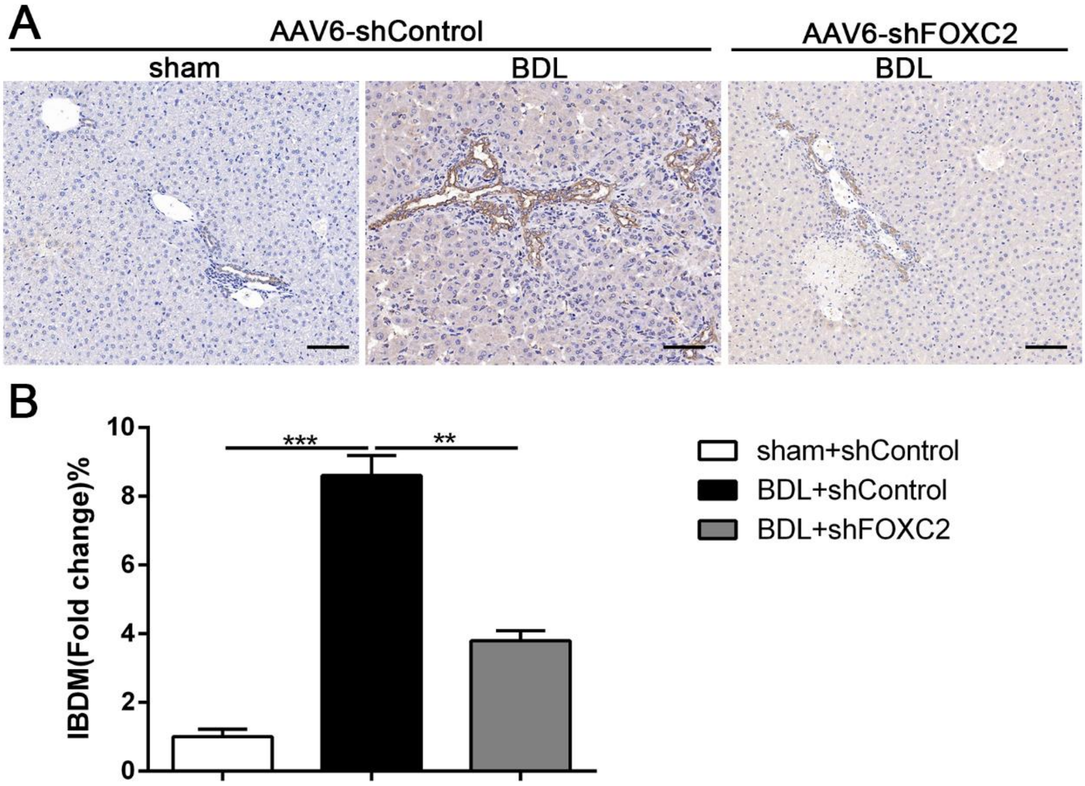

Figure S10 The cholangiocytes proliferated area in BDL model was assessed by IHC staining of CK-19. (A-B) IHC staining of CK-19 showed that targeting FOXC2 by *AAV6* shRNA partly abrogated the BDL-induced the proliferation of bile duct epithelium. IBDM (changes in intrahepatic bile duct mass). Magnification is ×10, and scale bars = 100 μm. n = 6/group. $^{**}P < 0.01$, $^{***}P < 0.001$ by ANOVA.
